# Supplementary material for: A New Phased-Array Magnetic Resonance Imaging Receive-Only Coil for HBO2 Studies
Source: Sensors (Basel). 2022 Aug 14;22(16):6076. doi: 10.3390/s22166076 (PMC9416538; doi:10.3390/s22166076)
Supplement: Supplementary file 1 [file sensors-22-06076-s001.zip › sensors-1815190-supplementary.pdf]

SUPPLEMENTARY MATERIALS

A NEW PHASED ARRAY MAGNETIC RESONANCE IMAGING RECEIVE ONLY COIL FOR HBO2 STUDIES

Azma Mareyam[1*,] Erik Shank[2*], Lawrence L. Wald[1], Michael Qin[3], and Giorgio Bonmassar[1, a)]

[1] A. A. Martinos Center for Biomedical Imaging, Massachusetts General Hospital, Harvard Medical School.

[2] Department of Anesthesia, Massachusetts General Hospital, Harvard Medical School.

[3] Office of Naval Research.

S1. Hyperbaric Chamber &MRI Safety Overview

All personnel should undergo MRI and HBOT safety training, which consists of viewing safety material (e.g., videos), understanding facility access policies and zones, specific orientation for HBOT/MRI, passing MRI safety test, orientation on MRI and HBOT rooms, and subject preparation, Emergency Procedure Training, Orientation on quench button use and the sprinkler system in case of fire in the chamber.

S1.1. Testing a Hyperbaric Chamber for Safety

The MRI environment presents potentially severe safety hazards for patients in a hyperbaric chamber near or inside an MRI system bore. Below we discuss the risks associated with the static magnetic field, gradient magnetic field, and radiofrequency fields (Table S1) that may vary significantly in different MRI systems and chambers and thus lead to a different risk profile. HBO2 has potential risks of catastrophic rapid decompression should the chamber's integrity be breached while under pressure and may result in harm or even death to the patient. Additionally, hyperoxia in the chamber can cause a fire as oxygen supports and lowers the threshold for materials ignition and combustion.

The risks of hyperbaric oxygen therapy to patients are related to ambient pressure and hyperoxia elevation. They include seizures, barotrauma (typically to ears with incomplete equilibration but could consist of pneumothorax from incomplete equilibration in portions of a lung), and theoretically changes in the eye's curvature (myopia). Other risks include Eustachian tube blockage, ear problems, seizure disorder, inability to equilibrate ears during pressure changes (e.g., air travel), pregnancy, and untreated pneumothorax. Finally, claustrophobia or anxiety disorders are common risks and exclusion criteria for both HBO2 and MRI. We will describe the potential risks associated with each MR imaging component, as this may be considered the more complex technology from an FDA regulatory perspective.

S1.2. Effects of the Static Magnetic Field

MRI systems have a strong magnetic field called B0 inside the bore. However, the displacement force on the chamber's ferromagnetic materials accidentally left in the HBO2 chamber is caused by the spatial field magnetic gradient is highest at the bore entrance. Objects such as pens, maks, communication equipment, valves, and oxygen sensors that may contain ferromagnetic parts inside the chamber could fly due to the displacement forces or resist movement and may harm the patient. These hazards should be addressed for chambers, usually located in MRI zone I or accessible to the general public, and objects anticipated to enter MRI zone IV. The recommendation for large human-sized HBO2 chambers is to use polymers, glass, ceramics, or any other non-conductive and nonferromagnetic material and avoid metals altogether as a conservative acceptance criterion.

Furthermore, B0 induces a torque on magnetic materials, with the highest torque inside the bore where B0 is the strongest. Torques may induce unwanted displacement of objects in the chamber, with risks of harming the

patient or puncturing the chamber and risking a catastrophic rapid decompression.

Table S1. Potential adverse interactions between MRI environment and hyperbaric chamber [27].

Component	Physical Effect	General Hazards
Static Magnetic Field	Eddy Currents for moving conductive materials like fittings	Rapid decompression during chamber insertion due to B_0-induced torque.
	Acceleration due to magnetic force	Serious injuries or death due to the "missile effect" of objects over due to B_0-induced forces/torques.
Gradient Magnetic Field	Faraday's Induced Currents dB/dt<br>Eddy Currents for conductive materials	Chamber malfunction or rapid decompression due to induced vibrations or heat.<br>Patient's hearing damage due to induced vibrations. Chamber sensor malfunction due to currents or voltages.
Radio Frequency Field	Eddy Currents for conductive materials	Heating of metallic fixtures that may damage or produce a rapid decompression.
	RF arcing	Fire in the chamber.

S1.3. Effects of the Gradient Magnetic Field

The pulsed gradient magnetic fields used in MRI give rise to a time-varying magnetic field and electrical currents in the human body. Major concerns with the pulsed gradient fields are biological effects such as cardiac excitation and peripheral nerve stimulation [28,29]. These have been substantiated by reports of painful stimulation and perception of light flashes (magnetophosphenes). Current FDA guidance limits the Time Rate of Change of Magnetic Field (dB/dt) to levels that do not result in painful peripheral nerve stimulation. Unpleasant or painful stimuli can be hazardous for many reasons, and, most of all, the subject's reaction under such stress could trigger sudden, jerky movements in the chamber [30–32]. The safety margin between perception and pain thresholds for a large subject is a factor of 2.6-4.0 for a sagittal or 3.9-6.0 for a longitudinal exposure [33]. This is a relatively small safety margin factor. Therefore, special care must be taken while testing the chamber on humans [34], and initially, the chamber should be tested at just slightly over 1 ATA. This will allow the operator to immediately stop the scanning and extract the subject from the chamber in case of an adverse event. Furthermore, voltages or currents through devices inside the chamber may produce malfunction or wrong readings, such as pressure gauges and temperature sensors.

The MR system's pulsed gradient magnetic fields, dB/dt, may induce forces on metallic fitting (e.g., copper) that result in vibrations. This gradient-

induced vibration of the chamber or only fittings may lead to catastrophic rapid decompression or chamber damage. Vibrations may also result from direct contact between the chamber and bore, especially for MRI sequences, such as diffusion tensor imaging (DTI), that produce significant vibrations.

S1.4. Effects of the Radio Frequency Field

The radiofrequency (RF) and time-varying gradient fields (dB/dt) of the MR system can induce heating of the chamber's metals, including the fittings. Metallic loops, commonly part of oxygen delivery masks or air hoses connectors, are mainly a fire hazard, and the power deposited is primarily determined by the relative orientation of the metallic loops with respect to the RF field. This hazard should be addressed for all objects anticipated to enter the bore of the MRI system. Careful screening by a radiologist to check if patients contain any ferrous metal (includes surgical clips, iron fragments from industrial sources, metal from weapons (e.g., bullet fragments), any implantable electronics (pacemakers, defibrillators, vagal nerve or brain stimulators, etc.), any wires (e.g., pacemaker wires), or any surgically implanted prosthesis or joint, that cannot be verified not to be non-conductive. It is generally accepted [35] that the Specific Absorption Rate (SAR) is the widely adopted dosimetry for determining the RF safety of MRI. The SAR studies are critical at frequencies above 1 MHz, in which heating of skin tissues occurs before peripheral nerve stimulation by electrical currents [28,36]. The SAR limits prescribed by the FDA are designed to limit the induced currents in the biological tissue below the threshold of adverse biological response. This response reflects any biochemical change, functional impairment, or pathological lesion that could impair performance and reduce the ability of an organism to respond to the additional challenge. Thus far, there are only extremely rare incidents [37,38] involving MRI scans within the described limits of SAR.

S1.5. Special considerations for RF and Electrostatic arcing

Arcing is a change of neutral gas from an insulation state to a conducting state, an insulation breakdown that will occur at a particular electrical field E. Generally, the voltage at which the arcing occurs is defined as the dielectric strength of the gas. Breakdown in gases is generally produced by impact ionization of the gas molecules at DC, while it is produced by electron diffusion at RF [39]. Arcing poses a high fire risk in the HBO2 chamber, which must be mitigated at all costs since a chamber fire in a hyperoxic environment can rapidly be fatal. RF arcing due to the high power and voltages in the coil can occur if there is no mitigation. RF arcing can be mitigated by clean soldering, careful RF component placement, adding fuses, potting to avoid air pockets near high voltage points, and thorough testing. Electrostatic arcing occurs to build up charges on dry polymers and can be mitigated by allowing for at least 40-60% of humidity in the chamber.

S1.6. Image artifacts

The presence of metallic parts inside or part of the chamber (e.g., metallic fittings) or other objects (e.g., metallic components of the breathing mask) can lead to artifacts in the acquired MR images. If the chamber is made of polymers that can absorb moisture (e.g., acrylic), the chamber itself can become quite conductive (e.g., at 128 MHz for 3T) and may absorb RF and reduce the SNR of the images. Furthermore, materials that are part of or in the chamber (e.g., metallic fittings) may produce artifacts due to Eddy currents at RF frequency or susceptibility artifacts due to material properties (para or diamagnetic). Thus, the operation of an HBO2 chamber may lead to artifacts or corruption of the acquired MR images. Both can lead to uninterpretable or non-diagnostic images or disease-mimicking artifacts. However, the various changes in gas concentration are not likely to cause artifacts as the electrical and magnetic properties of air and oxygen are very similar (i.e., air $\varepsilon_r = 1.0005364$ and $\chi_v = 3.73 \cdot 10^{-7}$ while for oxygen $\varepsilon_r = 1.0004947$ and $\chi_v = 3.6 \cdot 10^{-7}$).

S1.7. Special considerations for functional magnetic resonance imaging (fMRI)

Carbon dioxide (CO2) is a potent vasodilator that affects cerebral metabolism and thus the fMRI signal. Therefore, airflow needs to be strictly controlled to avoid hypercapnia potentially resulting from breathing masks. Notably, hypercapnia is routinely used in calibrated blood-oxygen-level-dependent (BOLD) or fMRI signals to estimate cerebral oxygen metabolism changes (ΔCMRO2). However, this calibration relies on the assumption that changes in ΔCMRO2 are small compared to the increases in hypercapnia-induced cerebral blood flow (CBF) [40,41], which MRI measurements can also infer. The recommendation is to ventilate the chamber with an airflow exceeding minute ventilation during an fMRI scan, thus preventing accumulating exhaled carbon dioxide.

Bench measurements showed the S-parameters of the RF-coil during coil constructions.

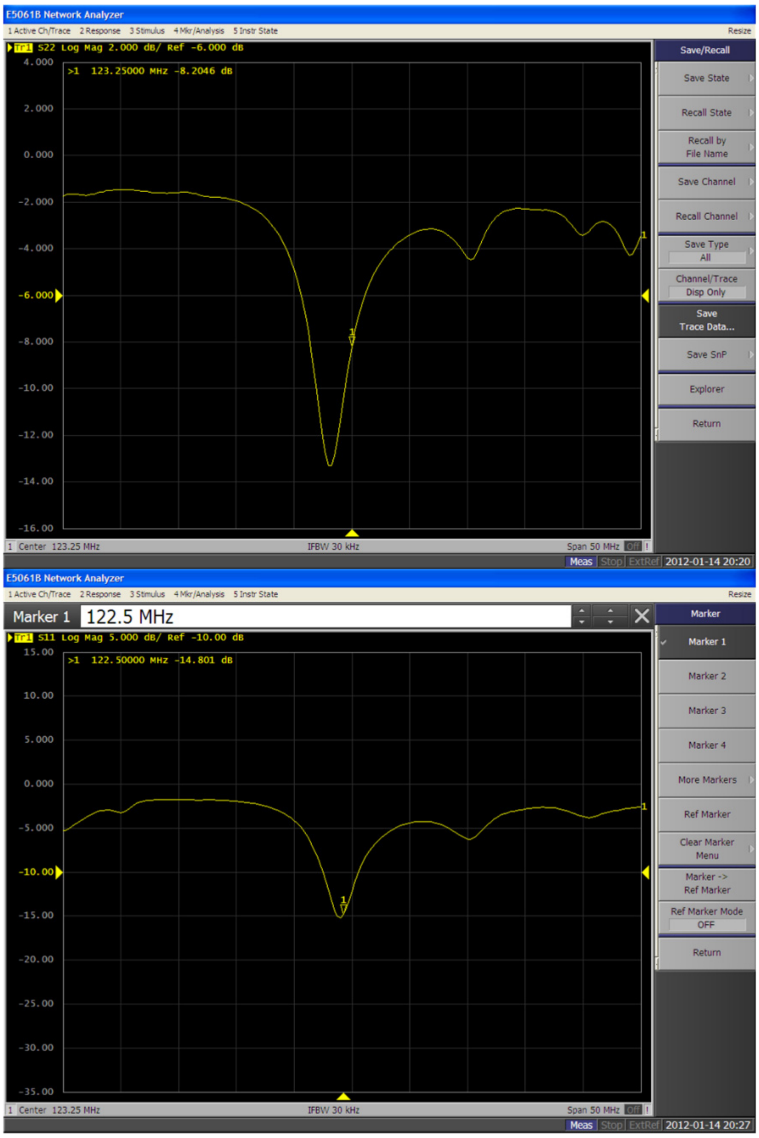

Figure S1. shows the reflection parameter S_{11} measured at 123.25 MHz (Larmor frequency of a 3 Tesla MRI system) of one of the eight coil elements unloaded, which is -8.3 dB and when loaded with a head is matched to a 50Ω loaded impedance. The load was not present (Top) or was a volunteer (bottom), which was slightly detuned (122.5MHz), given the original load was a phantom.

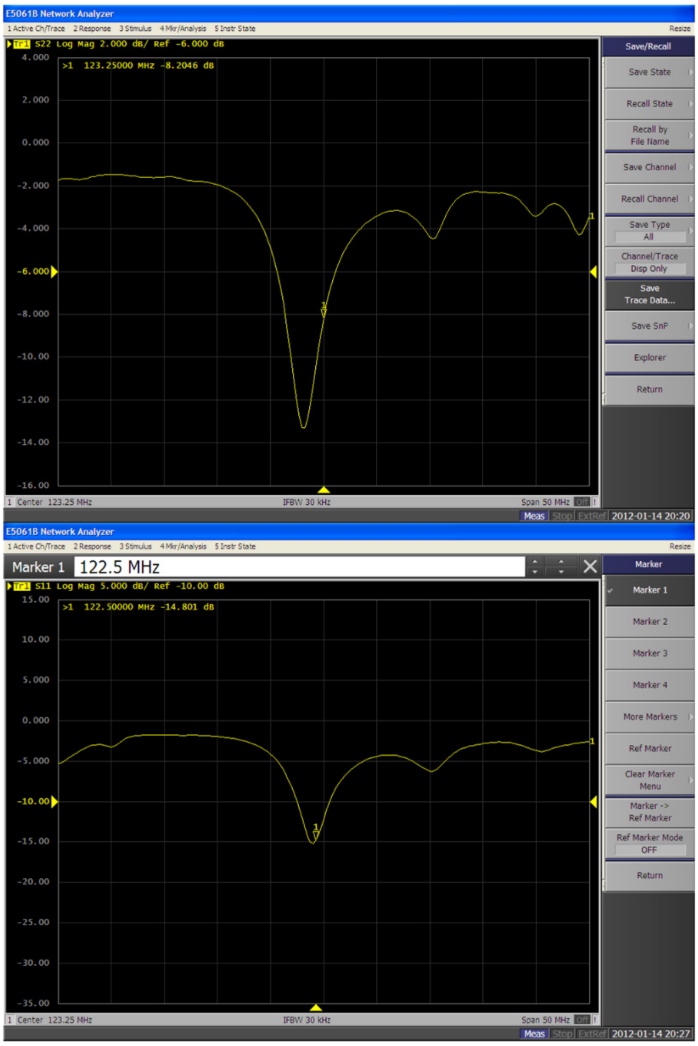

Figure S2. shows the reflection parameter S_{22} at 123.25 MHz of one of the overlapped neighboring coil elements to Figure S1, which shows a similar impedance to its neighbor (Figure S1). The load was not present (Top) or was a volunteer (bottom), which was slightly detuned (122.5MHz) given the original load was a phantom.

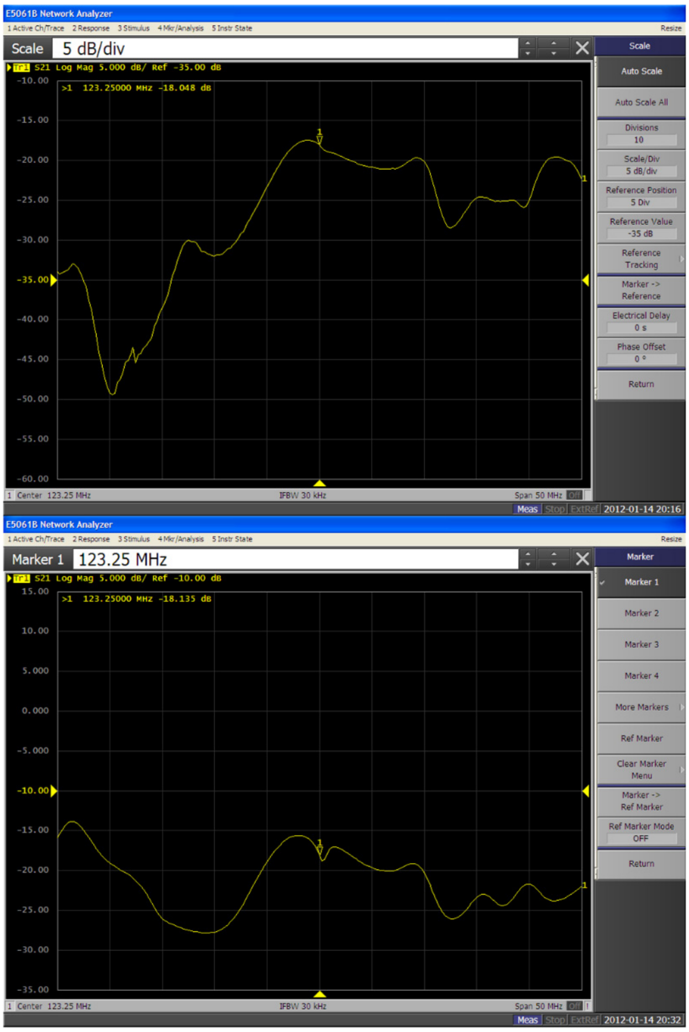

Figure S3. shows the transmission parameters S_{21} at 123.25 MHz between the two overlapped neighboring coil elements, measuring -18dB, which demonstrates that the two neighboring coil elements are decoupled from each other. The load was not present (Top) or was a volunteer (bottom), which was slightly detuned (122.5MHz), given the original load was a phantom.

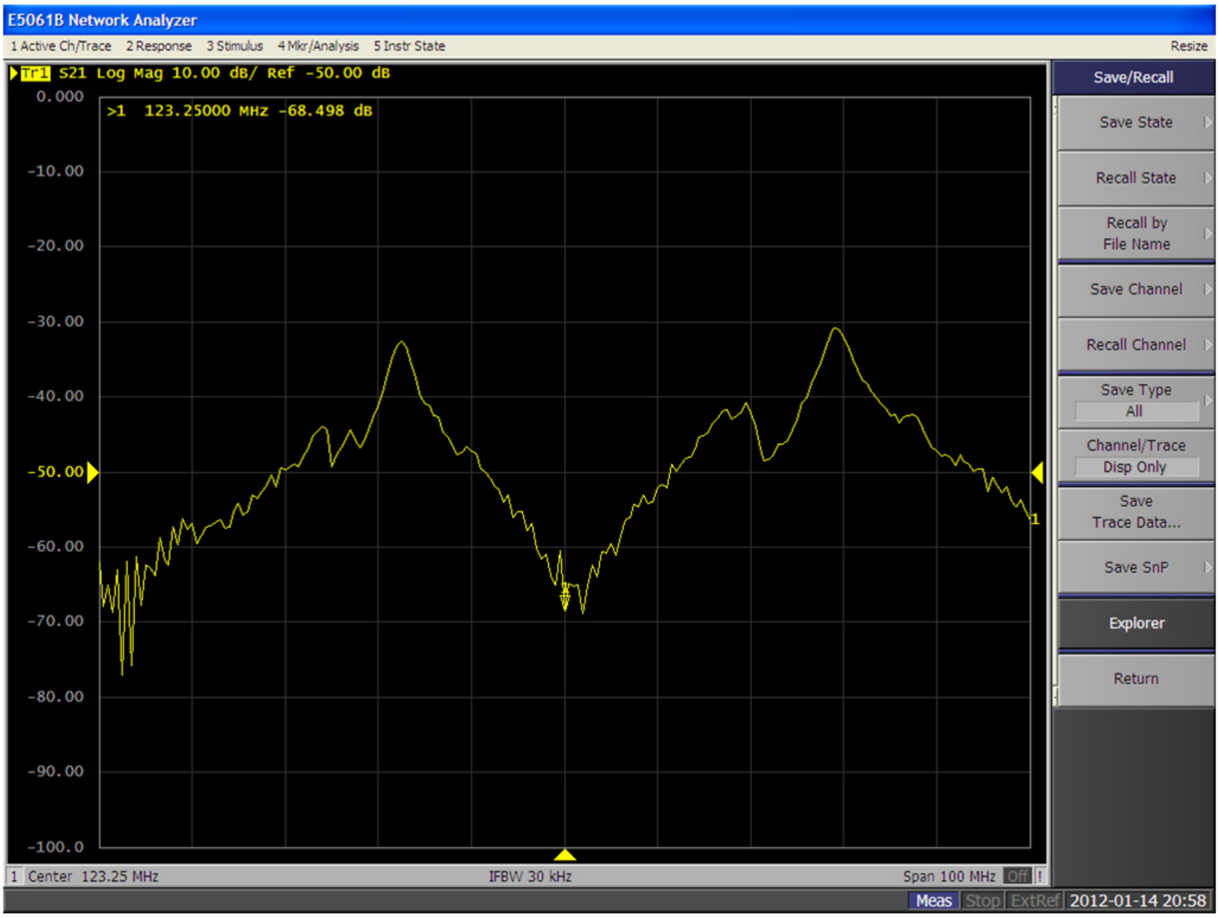

Figure S4. shows the typical detuning waveform at 123.25 MHz for each of the eight-receive coil elements with a detuning parameter of -68 dB, demonstrating that each receive coil element is detuned or turned OFF during transmission with the MRI body coil.

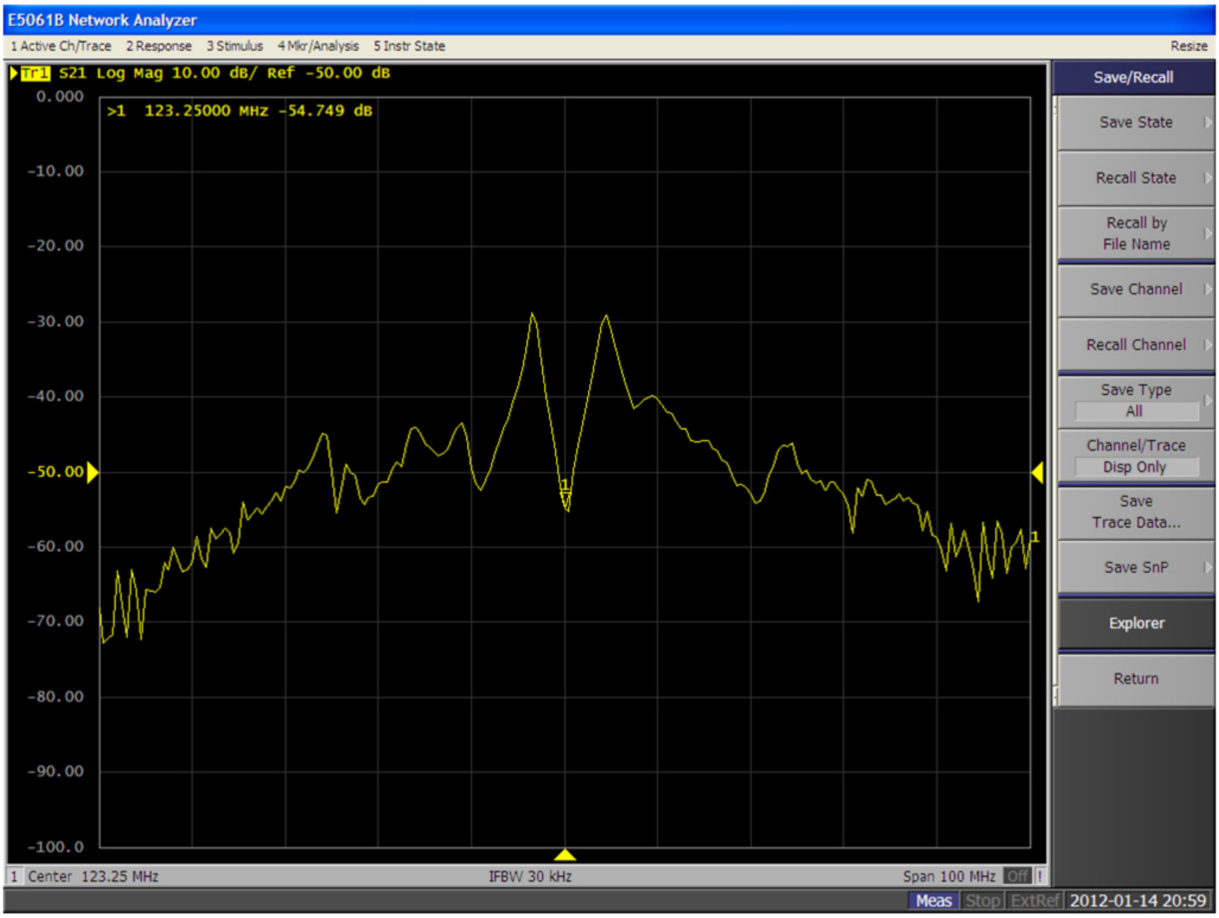

Figure S5. shows the typical preamp decoupling waveform at 123.25 MHz for each of the eight-receive coil elements providing additional decoupling between the receive coil elements.

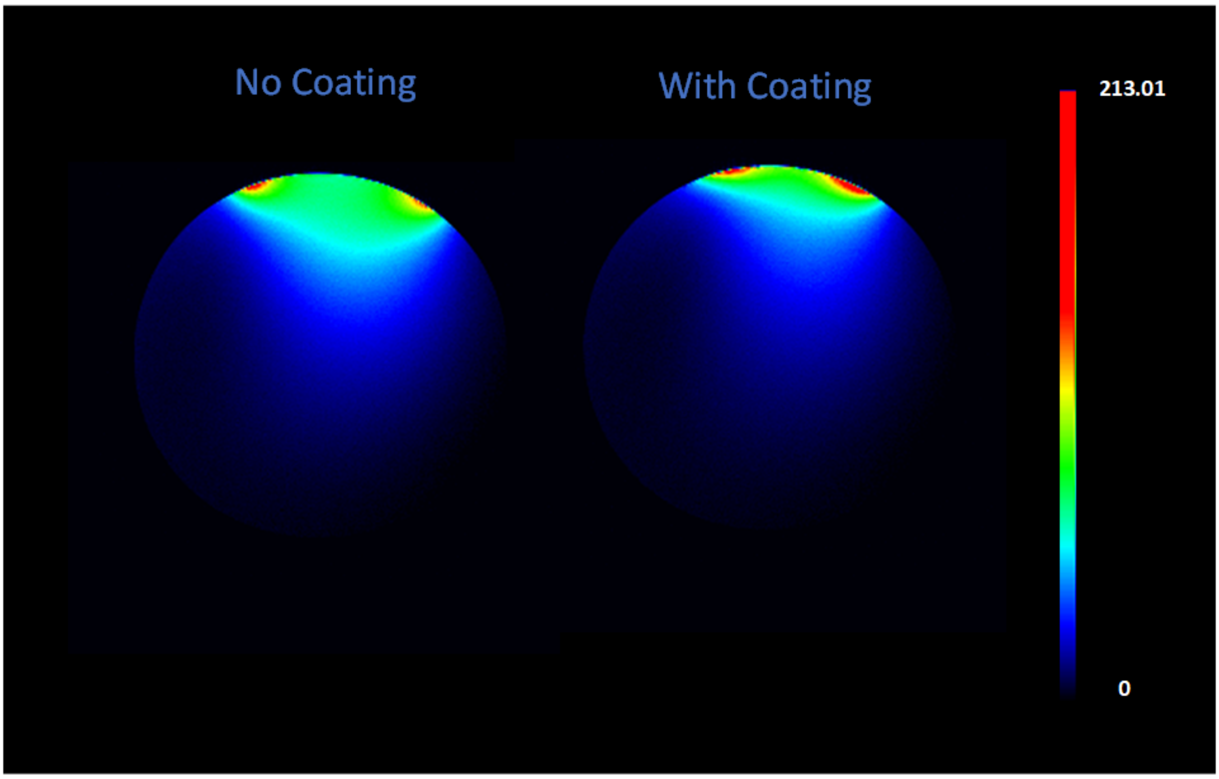

Figure S6: shows that coating does not reduce the SNR of the coil. Normobaric 3 Tesla MRI Signal to Noise Ratio tests of a saline phantom with a single channel coil (OD=9cm): (Left) with no coating and (right) with coating.
